# Multi-Strain Probiotic Improves Tryptophan Metabolism and Symptoms in Chronic Fatigue Syndrome Patients with Co-Occurring Irritable Bowel Syndrome: An Open-Label Pilot Study

**DOI:** 10.3390/nu18010174

**Published:** 2026-01-05

**Authors:** Cezary Chojnacki, Marta Mędrek-Socha, Jan Chojnacki, Anita Gąsiorowska, Ewa Walecka-Kapica, Michal Bijak, Karolina Przybylowska-Sygut, Tomasz Poplawski

**Affiliations:** 1 Department of Clinical Nutrition and Gastroenterological Diagnostics, Medical University of Lodz, 90-647 Lodz, Poland; cezary.chojnacki@umed.lodz.pl (C.C.);; 2 Department of Gastroenterology, Medical University of Lodz, 92-213 Lodz, Poland; anita.gasiorowska@umed.lodz.pl (A.G.);; 3 Biohazard Prevention Centre, Faculty of Biology and Environmental Protection, University of Lodz, Pomorska 141/143, 90-236 Lodz, Poland; michal.bijak@biol.uni.lodz.pl; 4 Department of Pharmaceutical Microbiology and Biochemistry, Medical University of Lodz, 92-215 Lodz, Poland

**Keywords:** Chronic Fatigue Syndrome, tryptophan metabolism, kynurenine pathway, gut microbiota, probiotics, quinolinic acid, leaky gut

## Abstract

**Background/Objectives**: Gut dysbiosis in Chronic Fatigue Syndrome (CFS) drives low-grade inflammation and shifts tryptophan metabolism toward neurotoxic pathways. The causal link between bacterial translocation, kynurenine pathway dysregulation, and symptom severity remains under-defined. We evaluated the impact of a high-concentration multi-strain probiotic on the “gut-kynurenine axis” and clinical status in CFS patients with co-morbid IBS-U and confirmed dysbiosis. **Methods**: Forty female patients with confirmed dysbiosis (GA-map™ Dysbiosis Index > 2) received the CDS22 formula (450 billion CFU/day) for 12 weeks. We compared urinary tryptophan metabolite profiles (LC-MS/MS), gut dysbiosis markers (3-indoxyl sulfate), and fatigue severity (FSS) against 40 age-matched healthy controls. **Results**: Baseline analysis revealed profound metabolic perturbations: elevated bacterial proteolytic markers (3-IS), substrate depletion (low tryptophan), and a neurotoxic signature (high quinolinic acid [QA], low kynurenic acid [KYNA]). Following the intervention, fatigue scores declined by 40.3%, with 97.5% of patients reaching the remission threshold (FSS < 36). Biochemically, 3-IS levels decreased to the range observed in healthy controls and attenuated xanthurenic acid levels. Although absolute QA concentrations remained elevated compared to controls, the neuroprotective KYNA/QA ratio increased significantly (+45%). Increased systemic tryptophan availability correlated directly with clinical symptom reduction (Spearman’s rho = −0.36, *p* = 0.024). **Conclusions**: The CDS22 formulation was associated with a restoration of intestinal eubiosis and functional tryptophan partitioning. Clinical remission coincides with a metabolic shift favoring neuroprotection (increased KYNA/QA ratio), validating the gut–kynurenine axis as a modifiable therapeutic target. Peripheral metabolic improvement relative to the healthy baseline appeared sufficient for symptom relief in this specific phenotype, despite incomplete clearance of neurotoxic metabolites.

## Simple Summary

Chronic Fatigue Syndrome (CFS) is a debilitating condition often accompanied by gut health issues, but effective treatments are scarce. Recent research suggests that an imbalance in gut bacteria (dysbiosis) may contribute to CFS symptoms by producing harmful substances that affect the nervous system. We investigated whether a specific multi-strain probiotic (CDS22-formula) could improve symptoms in women with CFS and co-occurring IBS. Over 12 weeks, patients took a high-dose probiotic supplement. We monitored their fatigue levels and analyzed urine samples to track changes in tryptophan metabolism—a key pathway linking the gut to the brain. The results showed that the probiotic intervention was associated with an improved gut bacteria profile. Importantly, this coincided with a reduction in neurotoxic metabolites and a significant decrease in fatigue severity. Our findings suggest that targeting the gut microbiome can be a valuable strategy for managing chronic fatigue, potentially by modulating the production of metabolites that affect brain function.

## 1. Introduction

Chronic fatigue manifests across various pathologies but serves as the primary, sometimes isolated, symptom of Chronic Fatigue Syndrome (CFS) [1,2]. Characterized by persistent or recurrent exhaustion, the syndrome’s diagnostic criteria have undergone repeated modification [3,4]. While historical classifications framed CFS as a psychosomatic or inflammatory disorder [5], recent investigation has shifted toward gut microbiome alterations (dysbiosis) [6,7]. Despite this focus, a definitive causal relationship remains elusive [8,9].

Data regarding specific bacterial populations are inconsistent; studies have reported increases in both pro- and anti-inflammatory strains [10,11,12]. A prevailing hypothesis posits that low-grade intestinal inflammation compromises barrier integrity, facilitating the translocation of bacterial and dietary antigens into the systemic circulation [13,14,15]. Elevated serum antibacterial antibodies and pro-inflammatory cytokines in CFS patients support this inflammatory model [16,17], yet evidence linking specific dysbiotic profiles to pathogenesis remains mixed.

“Dysbiosis” is traditionally defined by quantitative shifts in bacterial composition, diversity, or the ratio of commensal to pathogenic species [18,19,20,21]. However, taxonomic profiles do not always predict functional output [22]. As indicated by our previous study [23], structural microbiome changes often fail to correlate with metabolic activity. While metagenomic assessments in CFS patients yield conflicting results—some showing species overgrowth, others depletion—metabolic markers paint a more consistent picture. Independent of specific bacterial abundance, metabolic assessments (hydrogen/methane breath tests) and urinary markers (hippuric acid, indoxyl) frequently indicate functional bacterial overgrowth [23]. Crucially, this metabolic shift coincides with the overproduction of neurotoxic tryptophan metabolites, specifically xanthurenic and quinolinic acid. Since gut dysbiosis is a shared pathophysiological core linking enteric symptoms (IBS) with systemic neurotoxicity (CFS), we purposely recruited patients presenting with this overlapping phenotype to target the gut–brain axis directly.

These discrepancies between taxonomic structure and metabolic function necessitate a targeted therapeutic intervention. Probiotics, particularly those with anti-inflammatory properties, remain a primary treatment strategy [24,25]. This study investigates the association between a high-concentration, multi-strain probiotic intervention (CDS22 formula) and the modulation of neuroactive tryptophan metabolites in CFS patients with co-occurring IBS-U. Specifically, we aimed to determine whether restoring gut eubiosis correlates with a shift from neurotoxic to neuroprotective kynurenine pathway activity and a reduction in fatigue severity.

## 2. Materials and Methods

### 2.1. Participants and Study Design

**Figure 1:**
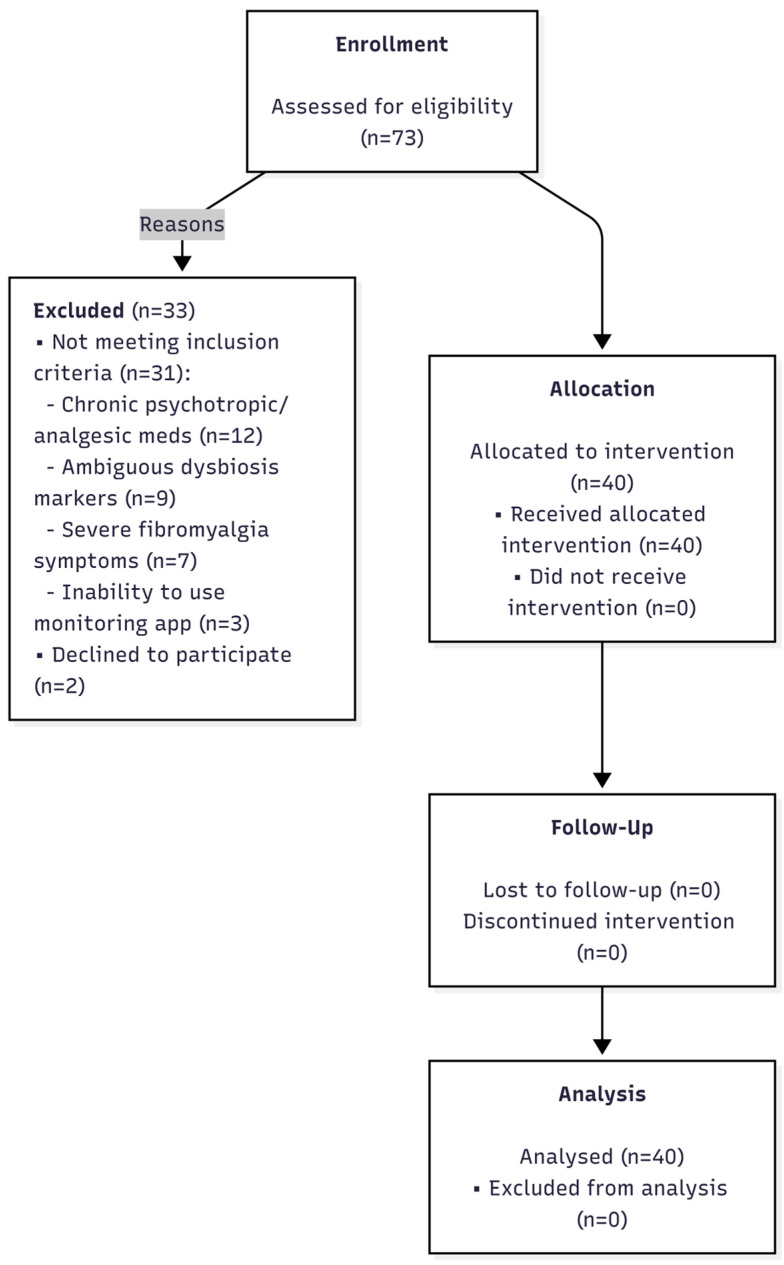
CONSORT flow diagram illustrating the screening, enrollment, and analysis of study participants.

A total of 73 patients presenting with overlapping symptoms of IBS and chronic fatigue were screened for eligibility at the Department of Gastroenterology outpatient clinic. Following detailed medical and laboratory evaluation, 33 candidates were excluded. The primary reasons for exclusion were chronic use of medications affecting the nervous system or gut motility (*n* = 12), ambiguous dysbiosis markers not meeting the threshold (*n* = 9), predominance of severe fibromyalgia pain (*n* = 7), and technical inability to comply with app-based monitoring (*n* = 3). Two additional patients declined participation. Ultimately, 40 female patients meeting all inclusion criteria were enrolled and completed the full 12-week intervention protocol. No dropouts or serious adverse events necessitating withdrawal were recorded. However, transient mild-to-moderate constipation and bloating were reported by 6 patients (15%) in the second week. These were successfully managed with temporary lactulose or macrogol supplementation (resolved within 3 days) without interrupting the probiotic regimen. The flow of participants through the study is depicted in Figure 1 (CONSORT Flow Diagram).

The study enrolled 40 female patients aged 29–56 years (mean 38.3 ± 7.1), recruited and treated between 2019 and 2025. The control group comprised 40 age-matched healthy women (mean 39.1 ± 11.3). Recruitment and diagnostic procedures were conducted at the Department of Gastroenterology and outpatient clinic of the Central Hospital of the Medical University in Lodz.

Patients presented with chronic fatigue, sleep disturbances, and abdominal symptoms persisting for 3 to 11 years. Inclusion required confirmed gut dysbiosis and tryptophan metabolism disorders [23] in the absence of abnormalities in routine laboratory profiles. Organic gastrointestinal, neurological, and psychiatric pathologies were ruled out via comprehensive laboratory and endoscopic evaluation, including histological assessment of duodenal and colonic mucosa.

Based on symptom variability and the lack of correlation between pain and defecation, patients were diagnosed with the unclassified subtype of irritable bowel syndrome (IBS-U) according to Rome IV Criteria [26,27]. Fatigue status and severity were quantified using the Chalder Fatigue Scale (CFQ-11) and the Fatigue Severity Scale (FSS), while dysbiosis was assessed using both indirect (Organix Gastro) and direct (GA-map Dysbiosis Test) methods alongside urinary tryptophan metabolite profiling.

Exclusion criteria included age > 55 years; inflammatory diseases of the gastrointestinal tract; hepatic, pancreatic, renal, or thyroid dysfunction; lactose or gluten intolerance; and malnutrition or obesity. Patients using hormonal contraceptives, probiotics, or psychotropic medications were also excluded.

### 2.2. Fatigue Assessment

The severity of fatigue was evaluated using the Chalder Fatigue Scale (CFQ-11) [28]. This 11-item instrument assesses two dimensions of fatigue: physical (items 1–7, e.g., lack of energy, need for rest, muscle weakness) and mental (items 8–11, e.g., difficulty concentrating, memory slips, finding words). Responses were recorded using a Likert scoring system (0–3), yielding a total score range of 0–33.

The impact of fatigue on daily functioning was quantified using the Fatigue Severity Scale (FSS) [29]. This 9-item questionnaire evaluates the interference of fatigue with physical activity, motivation, and social obligations. Participants rated each item on a 7-point scale (1 = strongly disagree, 7 = strongly agree). The total score ranges from 9 to 63, with higher scores indicating greater functional impairment. Both instruments were administered at baseline to confirm eligibility for the IBS-CFS group.

### 2.3. Laboratory Tests

Baseline biochemical and serological assessments were performed to exclude confounding metabolic, endocrine, or inflammatory pathologies. The screening panel included hematology (hemoglobin, HbA1c); renal function (urea, creatinine); liver function (bilirubin); and electrolytes (magnesium, potassium). Nutritional and hormonal status was assessed via serum iron, vitamin D3 (25-OH-D), vitamin B12, folic acid, TSH, fT3, fT4, FSH, and 17-β-estradiol. Celiac disease was ruled out using tissue transglutaminase (anti-tTG) and deaminated gliadin peptide (anti-DGP) antibodies. Systemic and intestinal inflammation was evaluated via C-reactive protein (CRP) and fecal calprotectin, respectively.

Urinary analysis focused on quantification of bacterial and tryptophan metabolites. First-morning void urine samples were collected on an empty stomach into sterile containers stabilized with 0.1% hydrochloric acid.

Gut microbial metabolites were assessed using the Organix Gastro profile (ALAB Laboratories, Warsaw, Poland). This panel quantified 11 organic acids, including p-hydroxyphenylacetic acid (HPA), hippuric acid (HA), and 3-indoxyl sulfate (3-IS), which serve as markers of intestinal bacterial overgrowth. Analysis was performed using gas chromatography–tandem mass spectrometry (GC-MS/MS).

Tryptophan metabolism was evaluated using the Organix Neuro panel (ALAB Laboratories). Concentrations of tryptophan (TRP) and its downstream metabolites—5-hydroxyindoleacetic acid (5-HIAA), kynurenine (KYN), kynurenic acid (KYNA), xanthurenic acid (XA), and quinolinic acid (QA)—were determined via liquid chromatography–tandem mass spectrometry (LC-MS/MS) utilizing an Agilent Technologies liquid chromatograph (Agilent Technologies, Santa Clara, CA, USA) coupled to a Waters mass spectrometer (Waters Corporation, Milford, MA, USA). Data acquisition and processing were conducted using Analyst 1.5.2 software.

Urine samples were collected during the follicular phase at baseline and after 12 weeks of probiotic intervention. Metabolite concentrations were normalized to urinary creatinine and expressed as milligrams per gram of creatinine (mg/gCr). To ensure pre-analytical validity, participants observed a 4-week washout period for antibiotics and probiotics and restricted dietary tryptophan intake to 15 mg/kg/day for three days prior to sampling.

### 2.4. Direct Dysbiosis Assessment (GA-Map™ Technology)

Gut microbiota composition and the degree of dysbiosis were assessed using the standardized, commercially available GA-map™ Dysbiosis Test (Genetic Analysis AS, Oslo, Norway) [30]. All analyses were performed externally by a certified reference laboratory (ALAB Laboratories, Warsaw, Poland) employing the complete, licensed manufacturer’s platform (marketed locally by ALAB Laboratories as the FloraGen panel) (ALAB Laboratories, Warsaw, Poland).

The assay utilizes 54 DNA probes targeting variable regions (V3–V9) of the bacterial 16S rRNA gene. These probes specifically identify over 300 bacterial taxa across six phyla: Actinobacteria, Bacteroidetes, Firmicutes, Proteobacteria, Tenericutes, and Verrucomicrobia. Hybridization was performed on the Luminex MAGPIX platform (Luminex Corporation, Austin, TX, USA).

Raw data processing and dysbiosis calculation were conducted exclusively via the GA-map™ Analyzer Software v1.4. This software employs a proprietary, closed-source algorithm developed and validated by Genetic Analysis AS to generate the Dysbiosis Index (DI). The study authors played no role in the development, modification, or calculation of this algorithm. The DI is a categorical score ranging from 1 to 5, defined against a normobiotic reference population:

DI 1–2: Normobiosis (non-dysbiotic profile).

DI > 2: Dysbiosis (3 = mild, 4 = moderate, 5 = severe).

Shannon alpha diversity was automatically calculated by the same software platform to provide a standardized measure of microbial richness and evenness.

Fecal samples were collected by participants into sterile, pathogen-free containers on the morning of the scheduled diagnostic visit. In strict adherence to the GA-map™ manufacturer’s validation for stability, samples were stored at ambient temperature (20–22 °C) for no longer than 7 days prior to analysis. This pre-analytical protocol is certified for the preservation of microbial DNA integrity in this specific assay.

### 2.5. Probiotic Intervention Protocol

Following the confirmation of intestinal dysbiosis via both direct and indirect assessments, participants were enrolled in a targeted probiotic intervention. The study utilized the De Simone Formulation (DSF), marketed as Vivomixx^®^ (currently rebranded as the CDS22 formula, Mendes S.A., Lugano, Switzerland). This poly-biotic preparation contains 450 billion colony-forming units (CFU) of eight specific, live bacterial strains: *Streptococcus thermophilus* DSM24731, *Bifidobacterium breve* DSM24732, *Bifidobacterium longum* DSM24736, *Bifidobacterium infantis* DSM24737, *Lactobacillus acidophilus* DSM24735, *Lactobacillus plantarum* DSM24730, *Lactobacillus paracasei* DSM24733, and *Lactobacillus delbrueckii* subsp. *bulgaricus* DSM24734.

The preparation is devoid of gluten, lactose, and genetically modified organisms, holding both Generally Recognized As Safe (GRAS, USA) and Qualified Presumption of Safety (QPS, EU) status. Sachets were stored at 2–8 °C to ensure viability.

The study was conducted as a prospective, open-label clinical trial. Patients were administered one sachet (450 billion CFU) daily in the morning for a period of 12 weeks. Compliance and tolerability were monitored via dietary diaries submitted at weeks 4, 8, and 12. Clinical status was reassessed at these time points using the fatigue severity questionnaires. The primary biochemical endpoint—profiling of urinary tryptophan metabolites (Organix Neuro) and gut dysbiosis markers (Organix Gastro)—was evaluated at baseline and upon completion of the 12-week protocol.

### 2.6. Nutritional Control and Dietary Monitoring

To minimize the confounding impact of diet on microbiome composition and metabolite profiles, all participants followed a standardized nutritional protocol. Throughout the study, subjects maintained a habitual, normocaloric diet (approximately 2000 kcal/day) with a standardized macronutrient distribution (minimum daily intake: protein 50 g, carbohydrates 270 g, fat 70 g, and soluble fiber 30 g).

Strict pre-analytical dietary controls were implemented prior to biochemical testing. For three days preceding urine sample collection, dietary tryptophan intake was controlled and capped at 15 mg/kg body weight/day in both groups to reduce inter-individual variability in amino acid availability.

Adherence to dietary recommendations was verified through supervised dietary logs, reviewed regularly by trained dietitians via telephone and email. Nutrient intake, specifically amino acid content, was quantified using Kcalmar Pro-Premium software (Hermex, Lublin, Poland; 2024 version), which utilizes a database of over 2500 products and has been validated for nutritional assessment in Polish populations.

Patients received initial training on food logging and portion estimation. Data were reviewed weekly by certified clinical dietitians (M.M.-S., E.W.-K.) via standardized telephone consultations to ensure adherence to the macronutrient and tryptophan limits. Clinical assessments and fatigue questionnaires were administered by board-certified gastroenterologists (C.C., J.C., A.G.) who were blinded to the ongoing biochemical results. To ensure data validity and mitigate the limitations of self-reporting, entries were reviewed weekly by a clinical dietitian. Furthermore, the stability of urinary creatinine levels across time points served as an internal biochemical control, confirming consistent hydration status and dietary compliance during the intervention period.

### 2.7. Ethical Statement

The study was conducted as an open-label clinical trial in strict accordance with the Declaration of Helsinki and the International Council for Harmonisation Guidelines for Good Clinical Practice (ICH-GCP). Written informed consent was obtained from all participants prior to enrollment. The study protocol received formal approval from the Bioethics Committee of the Medical University of Lodz under resolutions RNN/176/18/KE (15 May 2018) and RNN/75/25/KE (15 April 2025).

Registration Statement: This study was not prospectively registered in a public clinical trial registry. Under Polish national law, clinical trial registration requirements—as defined by the Act of 9 March 2023 on Clinical Trials of Medicinal Products for Human Use (Ustawa z dnia 9 marca 2023 r. o badaniach klinicznych produktów leczniczych stosowanych u ludzi; Journal of Laws 2023, item 605) and EU Regulation No. 536/2014—apply exclusively to investigational medicinal products (produkty lecznicze). The intervention product used in this study (CDS22 formula, 450 billion CFU sachets) is officially registered as a dietary supplement (suplement diety) in the Register of Products Subject to Notification of First Marketing maintained by the Chief Sanitary Inspectorate (Główny Inspektorat Sanitarny, GIS) under entry No. 212378 (year 2025). As such, the study does not fall within the legal scope of clinical trial registration mandated for medicinal products. Official documentation from the GIS Register confirming the product’s classification as a dietary supplement has been provided to the Editorial Office. Despite the absence of a statutory registration requirement for dietary supplement intervention studies in Poland, the investigation was conducted with full ethical oversight and bioethics committee approval.

### 2.8. Statistical Analysis

All statistical analyses were performed using R software (version 4.5.1; R Foundation for Statistical Computing, Vienna, Austria) accessed via the RStudio integrated development environment (version 2025.09.2+418 “Cucumberleaf Sunflower”; Posit Software, PBC, Boston, MA, USA). Continuous variables are reported as median with interquartile range (IQR), and categorical variables as counts and percentages (%). Due to the non-Gaussian and skewed distribution of metabolite data, non-parametric tests were employed throughout.

#### 2.8.1. Hypothesis Testing and Effect Sizes

Between-group differences (baseline CFS vs. controls; post-treatment CFS vs. controls) were assessed using the Wilcoxon rank-sum test (Mann–Whitney U). Within-group changes (pre- vs. post-treatment) were evaluated using the paired Wilcoxon signed-rank test. Median differences are reported with Hodges–Lehmann estimates and 95% confidence intervals (CIs). To quantify the magnitude of observed differences, effect sizes were calculated for all comparisons:

- Cliff’s delta (δ) for independent samples.
- Rank–biserial correlation (r_rb_) for paired samples.
- 95% CIs for effect sizes were generated via bootstrap resampling (B = 4000 replicates).

Interpretation followed established thresholds: negligible (|δ| < 0.147; |r_rb_| < 0.10), small (0.147 ≤ |δ| < 0.330; 0.10 ≤ r_rb_ < 0.30), medium (0.330 ≤ |δ| < 0.474; 0.30 ≤ |r_rb_| < 0.50), and large (|δ| ≥ 0.474; |r_rb_| ≥ 0.50).

#### 2.8.2. Multiple Testing Correction

To control the family-wise error rate across the 13 primary metabolic endpoints (7 metabolites, 6 ratios), *p*-values were adjusted using the Benjamini–Hochberg False Discovery Rate (FDR) procedure. Adjusted q-values < 0.05 were considered statistically significant.

#### 2.8.3. Clinical and Longitudinal Analysis

Longitudinal Fatigue Severity Scale (FSS) scores (weeks 0, 4, 8, 12) were analyzed using the Friedman test, followed by Bonferroni-corrected paired Wilcoxon post hoc contrasts. Clinical response was defined as a ≥10-point reduction (Minimally Clinically Important Difference, MCID) or a decrease below the diagnostic threshold (<36 points).

Associations between metabolic shifts (Delta) and clinical improvement (Delta FSS) were assessed using Spearman’s rank correlation. Dose–response relationships were probed using the Jonckheere–Terpstra trend test across quartiles of tryptophan change (Delta TRP).

#### 2.8.4. Binary Outcomes

Changes in binary somatic symptoms were analyzed using McNemar’s test (with continuity correction or exact test for sparse data). Efficacy was quantified using Absolute Risk Reduction (ARR) and Number Needed to Treat (NNT). *p*-values for the four symptom endpoints were adjusted using the Holm method.

#### 2.8.5. Visualization

Data distributions were visualized using raincloud plots (combining half-violin, boxplot, and jittered raw data). Paired changes were depicted using patient-level trajectory plots and alluvial diagrams. Effect sizes were summarized in forest plots.

## 3. Results

### 3.1. Baseline Characteristics and Safety Profile

Baseline assessment confirmed significant gut microbiome alterations in the CFS cohort. While healthy controls exhibited normobiosis with a Shannon Diversity Index (SDI) of 3.19 ± 0.83, patients demonstrated a markedly elevated Dysbiosis Index (DI) of 3.72 ± 1.28 and reduced bacterial diversity (SDI 2.23 ± 0.51; *p* < 0.001).

Routine biochemical, renal, and hepatic profiles were largely within physiological reference ranges. A specific deficit was observed in serum iron levels, which were significantly lower in CFS patients compared to healthy controls at baseline (93.0 ± 11.1 vs. 109.8 ± 12.7 µg/dL; *p* < 0.05). Following the 12-week probiotic intervention, serum iron levels increased significantly (*p* = 0.046), effectively restoring values to the range observed in the control group. Other metabolic and endocrine parameters remained stable throughout the intervention, confirming the safety and tolerability of the preparation (Table 1).

### 3.2. Baseline Tryptophan Metabolite Profile Distinguishes CFS from Healthy Controls

#### 3.2.1. Overview of Metabolic Dysregulation

**Figure 2:**
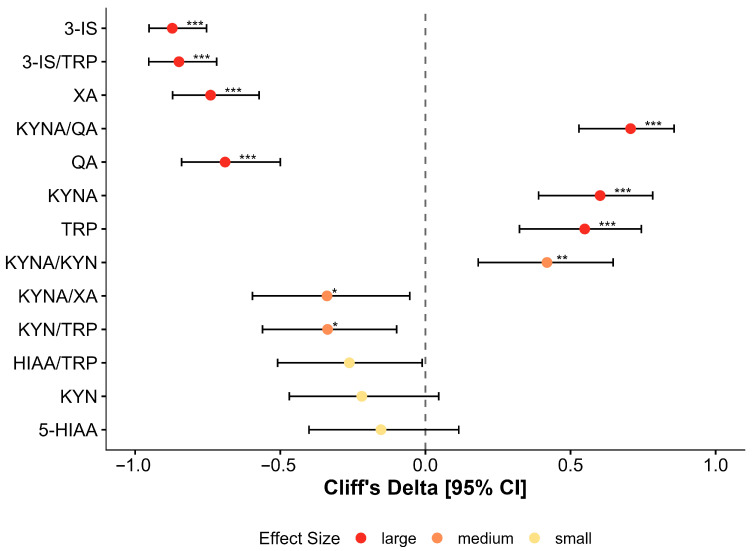
Baseline tryptophan metabolism: effect sizes for CFS patients vs. healthy controls. Forest plot displaying Cliff’s delta (δ) effect sizes with 95% bootstrap confidence intervals for 13 urinary tryptophan metabolites and ratios. Negative values indicate higher levels in CFS patients; positive values indicate lower levels compared to controls. Variables are ranked by statistical significance (Benjamini–Hochberg FDR). Colors indicate effect magnitude: red = large (δ ≥ 0.474); orange = medium; yellow = small. Note the substantial elevation in 3-indoxyl sulfate (3-IS) and quinolinic acid (QA) contrasting with the deficit in tryptophan (TRP) and kynurenic acid (KYNA). Asterisks indicate statistically significant differences (* *p* < 0.05; ** *p* < 0.01; *** *p* < 0.001).

Quantitative profiling of seven tryptophan metabolites and six metabolic ratios revealed profound baseline alterations in CFS patients. Following Benjamini–Hochberg False Discovery Rate (FDR) correction, 10 of 13 analyzed variables differed significantly from healthy controls (*q* < 0.05). Notably, seven of these variables exhibited large effect sizes (δ ≥ 0.474), indicating a robust and consistent metabolic shift (Figure 2, Table 2).

#### 3.2.2. Markers of Dysbiotic Tryptophan Catabolism

**Figure 3:**
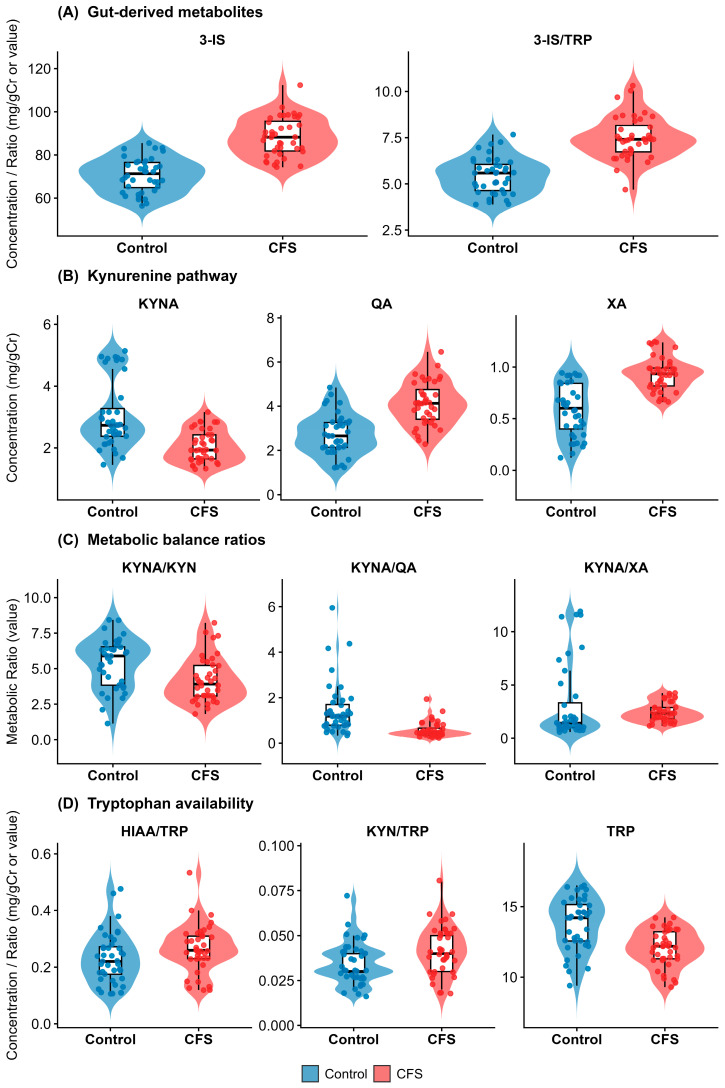
Baseline urinary metabolite distributions. Raincloud plots (combining probability density, boxplots, and jittered raw data) comparing CFS patients (red) and healthy controls (blue). (**A**) Gut-derived markers: elevated 3-IS in CFS indicates increased bacterial proteolysis. (**B**) Kynurenine pathway: elevated neurotoxic QA and reduced neuroprotective KYNA in CFS. (**C**) Metabolic ratios: the KYNA/QA ratio is markedly depressed in patients, reflecting a shift toward neurotoxicity. (**D**) Substrate availability: reduced TRP levels in the patient cohort.

Patients demonstrated a marked upregulation of bacterial tryptophan metabolism. Urinary 3-indoxyl sulfate (3-IS) was elevated by 23.8% in the CFS group compared to controls (median: 88.18 vs. 71.25 µmol/L; *p* < 0.001), with a large effect size (δ = −0.872). Similarly, the 3-IS/TRP ratio, reflecting the diversion of tryptophan toward bacterial indole pathways, was increased by 32.6% (*q* < 0.001). These findings corroborate the dysbiosis confirmed by the GA-map™ analysis (Section 3.1) (Figure 3A).

#### 3.2.3. Shift Toward Kynurenine Pathway Neurotoxicity

The kynurenine pathway exhibited a distinct pro-neurotoxic signature. Quinolinic acid (QA), a potent NMDA receptor agonist, was elevated by 55.8% in CFS patients (median: 4.13 vs. 2.65 µmol/L; *q* < 0.001). Conversely, the neuroprotective metabolite kynurenic acid (KYNA) was significantly reduced (1.93 vs. 2.74 µmol/L; *q* < 0.001). Xanthurenic acid (XA), a marker of vitamin B6-dependent kynurenine flux, was also elevated by 55.8% in the patient cohort (δ = −0.740; *q* < 0.001) (Figure 3B).

#### 3.2.4. Imbalance of Neuroprotective Capacity

The functional balance between neuroprotection and neurotoxicity was severely compromised. The KYNA/QA ratio was reduced by 60.5% in CFS patients compared to controls (median: 0.47 vs. 1.19; δ = 0.707; *q* < 0.001$). Furthermore, the efficiency of KYNA synthesis (KYNA/KYN ratio) was significantly lower (*q* = 0.002$), while the channeling of precursors toward xanthurenic acid (KYNA/XA ratio) was shifted in favor of XA (*q* = 0.012) (Figure 3C).

#### 3.2.5. Substrate Availability and Serotonin Pathway

Urinary tryptophan (TRP) availability was significantly lower in CFS patients (δ = 0.549; *q* < 0.001). Despite this substrate deficit, the activation of the kynurenine pathway relative to tryptophan (KYN/TRP ratio) was increased (*q* = 0.011), suggesting enhanced IDO/TDO enzymatic activity. The serotonin pathway marker, 5-HIAA, did not differ in absolute concentration, but the 5-HIAA/TRP turnover ratio showed a borderline significant elevation (*q* = 0.052) (Figure 3D).

### 3.3. Treatment-Induced Changes in Tryptophan Metabolism

#### 3.3.1. Overview of Treatment Response

**Figure 4:**
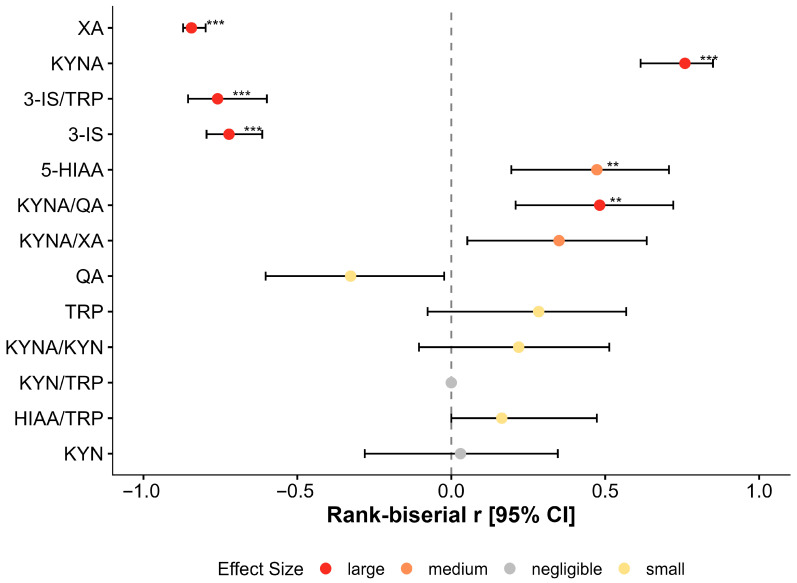
Magnitude of treatment effect (pre- vs. post-intervention). Forest plot of rank–biserial correlation (r_rb_) effect sizes for paired comparisons within the CFS cohort (*n* = 40). Negative values indicate a decrease post-treatment; positive values indicate an increase. The intervention produced large effects (r_rb_) ≥ 0.50) in normalizing xanthurenic acid (XA), 3-IS, and kynurenic acid (KYNA). Error bars represent 95% bootstrap confidence intervals. Asterisks indicate statistically significant differences (** *p* < 0.01; *** *p* < 0.001).

Following the 12-week probiotic intervention, paired analysis demonstrated significant metabolic improvements in 6 of the 13 evaluated variables (*q* < 0.05). Five of these changes were characterized by large effect sizes (r_rb_ ≥ 0.50), indicating a robust physiological response to therapy (Table 3, Figure 4).

#### 3.3.2. Attenuation of the Kynurenine Pathway

**Figure 5:**
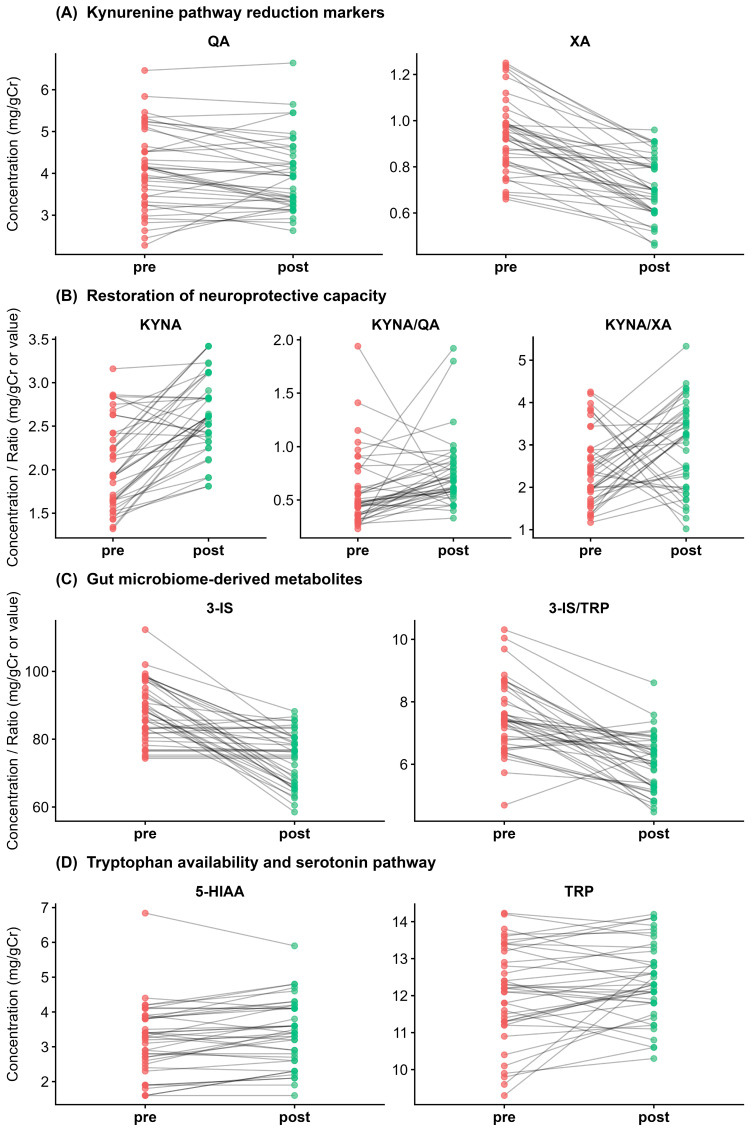
Individual patient trajectories during treatment. Slopegraphs connecting pre-treatment (red) and post-treatment (green) values for individual patients. (**A**) Consistent reduction in xanthurenic acid (XA) in 92.5% of patients. (**B**) Increase in neuroprotective KYNA and the KYNA/QA ratio. (**C**) Reduction in the dysbiosis marker 3-IS. (**D**) Tryptophan (TRP) levels show a heterogeneous response with a non-significant upward trend. The visualization highlights inter-individual variability in therapeutic response.

Treatment resulted in a marked downregulation of kynurenine pathway activation markers. Xanthurenic acid (XA) levels decreased by 25.1% (median: 0.94 to 0.70 mg/gCr), demonstrating a large effect size (r_rb_= −0.897; *q* < 0.001). Notably, this reduction was observed in 92.5% of patients (37/40). Quinolinic acid (QA) showed a nominal decrease (median: 4.13 to 3.92 mg/gCr; *p* = 0.038), but this change did not reach statistical significance after correction for multiple comparisons (*q* = 0.062) (Figure 5A).

#### 3.3.3. Restoration of Neuroprotective Capacity

A significant upregulation of the neuroprotective branch was observed. Kynurenic acid (KYNA) increased by 35.2% (median: 1.93 to 2.61 mg/gCr; *q* < 0.001), with 82.5% of patients showing an elevate response. Consequently, the neuroprotective-to-neurotoxic ratio (KYNA/QA) improved significantly (median: 0.47 to 0.68; r_rb_ = 0.590; *q* = 0.006), reflecting a shift toward a more favorable metabolic profile (Figure 5B).

#### 3.3.4. Reduction in Gut-Derived Metabolites

Markers of dysbiosis improved significantly. Urinary 3-indoxyl sulfate (3-IS) decreased by 13.5% (median: 88.18 to 76.25 mg/gCr; *q* < 0.001), and the 3-IS/TRP ratio declined by 17.8% (*q* < 0.001). These changes suggest a reduction in the bacterial catabolism of tryptophan within the gut lumen (Figure 5C).

#### 3.3.5. Serotonin Pathway and Substrate Availability

Levels of 5-HIAA increased significantly post-treatment (median: 3.28 to 3.40 mg/gCr; *q* = 0.006), indicating enhanced serotonin turnover. Tryptophan (TRP) availability showed a trend toward increase, but this was not statistically significant (*p* = 0.073; *q* = 0.105) (Figure 5D).

### 3.4. Post-Treatment Metabolic Profile Relative to Healthy Baseline

#### 3.4.1. Overview of Normalization Status

**Figure 6:**
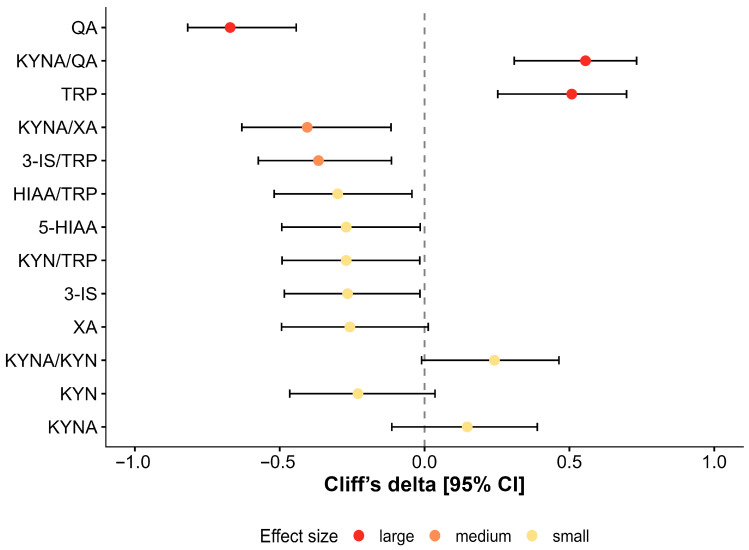
Comparative bio-profile: post-intervention CFS cohort vs. healthy reference group. Forest plot of Cliff’s delta comparing treated CFS patients with the healthy control baseline. Variables crossing the vertical zero line indicate statistical equivalence (normalization). Note that while 3-IS and XA normalized (small/negligible effects), QA and TRP retained large effect sizes, indicating incomplete resolution of these specific deficits. Also, this comparison illustrates the metabolic status of patients after treatment relative to the healthy population; it describes the degree of alignment with the reference range rather than placebo-controlled efficacy.

Comparison of post-treatment CFS patients (*n* = 40) with the healthy control baseline (*n* = 40) revealed a heterogeneous pattern of metabolic recovery. While 10 variables differed significantly at baseline, this number decreased to 6 post-treatment (*q* < 0.05). Crucially, the number of variables exhibiting large effect sizes (δ ≥ 0.474) dropped from seven to three, indicating substantial but incomplete restoration of metabolic homeostasis (Table 4, Figure 6).

#### 3.4.2. Complete Normalization of Gut and Kynurenine Markers

**Figure 7:**
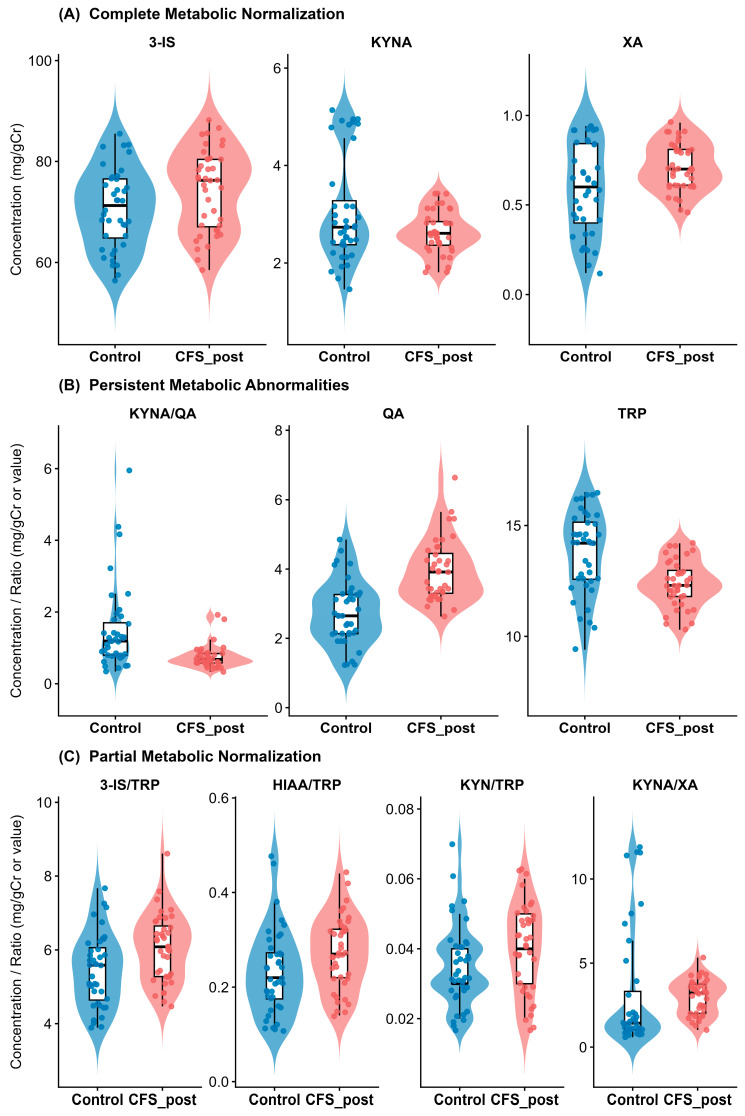
Distribution of tryptophan metabolites: treated patients relative to healthy baseline. Raincloud plots illustrating normalization status. (**A**) Statistical equivalence to controls: 3-IS and XA distributions in treated patients (red) overlap with healthy controls (blue). (**B**) Persistent deviation: QA remains elevated and TRP remains depressed despite treatment. (**C**) Intermediate shifts: ratios such as 3-IS/TRP show improvement but fail to reach full statistical equivalence with controls.

Several key metabolites achieved levels statistically indistinguishable from healthy controls. Urinary 3-indoxyl sulfate (3-IS), a marker of dysbiosis, was fully normalized (CFS post: 76.25 vs. controls: 71.25 mg/gCr; *q* = 0.060), reducing the effect size from large at baseline (δ = −0.87) to small/negligible (δ = −0.27). Similarly, xanthurenic acid (XA) returned to the control range (*q* = 0.062), suggesting successful attenuation of excessive kynurenine pathway flux. Kynurenic acid (KYNA) levels, initially depressed, were restored and showed no significant difference from the controls (*q* = 0.258) (Figure 7A).

#### 3.4.3. Persistent Neurotoxic Liability

Despite the improvements noted above, specific metabolic deficits remained refractory to treatment. Quinolinic acid (QA) remained significantly elevated in treated patients compared to controls (median: 3.92 vs. 2.65 mg/gCr; *q* < 0.001), retaining a large effect size (δ = −0.67). Consequently, the neuroprotective ratio (KYNA/QA) remained significantly lower than in healthy individuals (*q* < 0.001), indicating that the neurotoxic burden was reduced but not eliminated (Figure 7B).

#### 3.4.4. Persistent Tryptophan Deficit

Tryptophan (TRP) availability failed to normalize. Post-treatment levels in CFS patients remained significantly lower than in controls (median: 12.30 vs. 14.20 mg/gCr; *q* < 0.001; δ = 0.51), suggesting ongoing substrate depletion or malabsorption despite probiotic supplementation (Figure 7C).

### 3.5. Progressive Reduction in Fatigue and Symptom Resolution

#### 3.5.1. Fatigue Severity Scale (FSS) Trajectory

**Figure 8:**
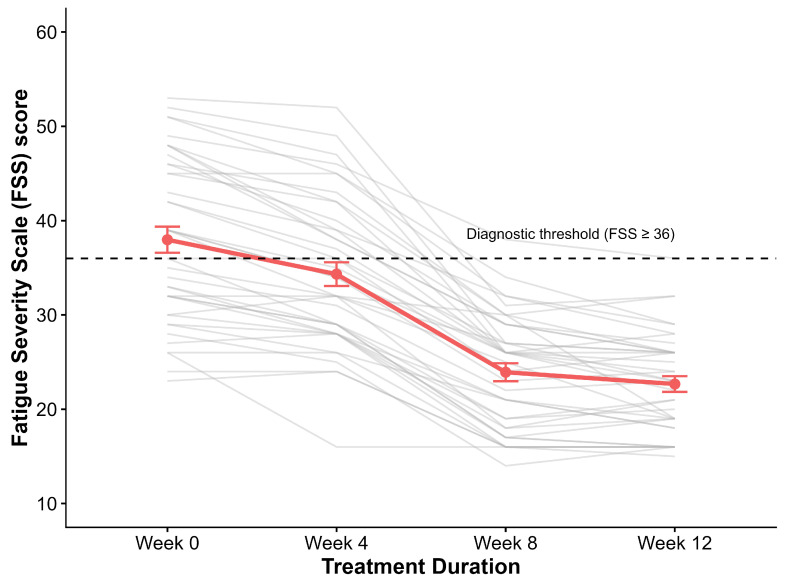
Progressive reduction in fatigue severity. Longitudinal trajectory of Fatigue Severity Scale (FSS) scores at baseline, week 4, week 8, and week 12. Gray lines represent individual patients; the red line indicates the group mean ± SE. The dashed line marks the diagnostic threshold (FSS < 36). Treatment resulted in a continuous, significant decline in fatigue (chi2 = 103.41, *p* < 0.001), with 97.5% of patients falling below the diagnostic threshold by week 12.

Probiotic intervention resulted in a continuous and highly significant reduction in fatigue severity across all time points (Friedman test: chi2 = 103.41, *p* < 0.001). The mean FSS score declined from 38.0 ± 8.8 at baseline to 22.7 ± 5.3 at week 12, representing a 40.3% reduction relative to baseline (Table 5). Post hoc pairwise comparisons confirmed significant improvements at weeks 4, 8, and 12 compared to baseline (all adjusted *p* < 0.001) (Figure 8).

#### 3.5.2. Clinical Response Rates

By week 12, 39 of 40 patients (97.5%) achieved the diagnostic threshold for remission (FSS < 36), compared to only 18 (45.0%) at baseline. A Minimally Clinically Important Difference (MCID), defined as a reduction of ≥10 points on the FSS, was achieved by 35 patients (87.5%).

#### 3.5.3. Somatic Symptoms and Tolerability

Gastrointestinal complaints, specifically bloating and abdominal pain, improved in the majority of participants. However, constipation showed a divergent pattern, worsening in 6 patients (15%) and necessitating temporary laxative use. Sleep disturbances were the most refractory symptom, persisting in the majority of the cohort despite the reduction in daytime fatigue. The probiotic preparation was otherwise well-tolerated (Table 6).

### 3.6. Tryptophan Availability Predicts Magnitude of Clinical Benefit

To investigate the biological basis of the observed clinical improvement, we analyzed the relationship between metabolic shifts and fatigue reduction. A significant negative correlation was identified between the increase in urinary tryptophan (ΔTRP) and the change in fatigue severity ΔFSS) (Spearman’s rho = −0.36, *p* = 0.024). This indicates that patients who experienced greater restoration of tryptophan availability achieved larger reductions in fatigue scores.

**Figure 9:**
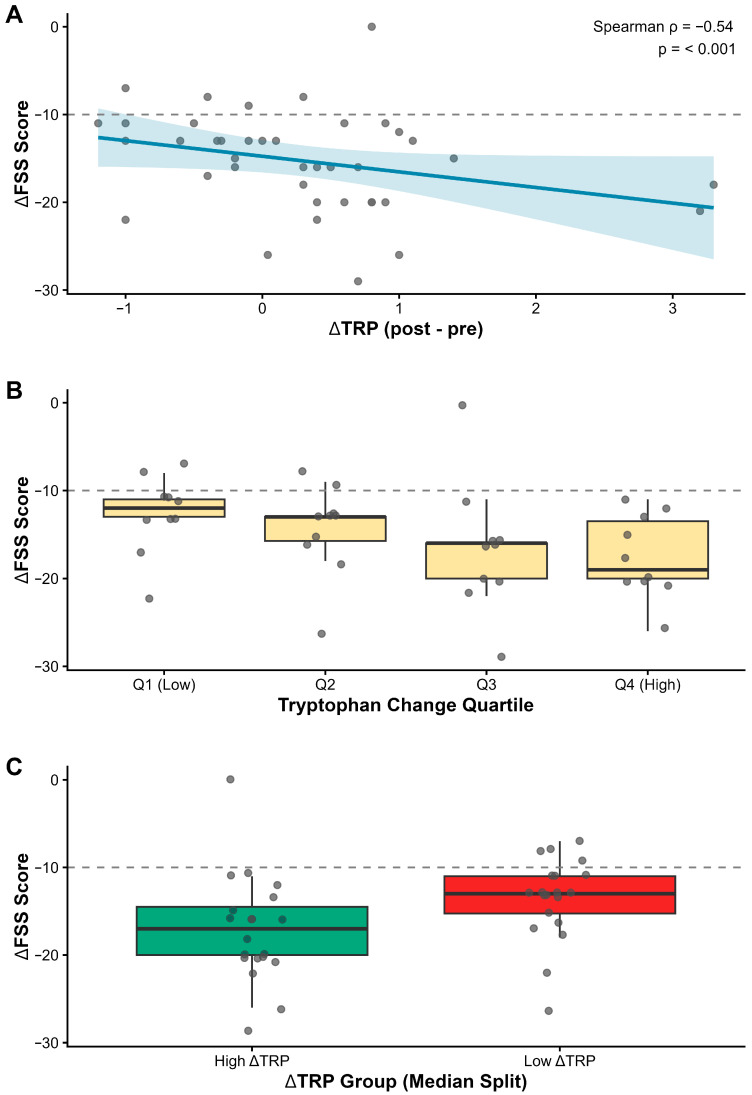
Tryptophan availability correlates with clinical improvement. (**A**) Scatter plot showing the relationship between the increase in urinary tryptophan (ΔTRP) and the reduction in fatigue severity (ΔFSS). The blue line represents the linear trend; the association is statistically significant (Spearman’s rho = −0.36, *p* = 0.024). (**B**) Boxplot of fatigue reduction stratified by quartiles of tryptophan change, demonstrating a monotonic dose–response relationship (*p*_{trend} = 0.007). (**C**) Patients with above-median tryptophan increases achieved significantly greater fatigue reduction than those with below-median changes.

This relationship followed a monotonic dose–response pattern (Jonckheere–Terpstra trend test, *p* = 0.007). Patients in the highest quartile of tryptophan increase (ΔTRP > median) exhibited significantly greater clinical improvement compared to those in the lowest quartile (median ΔFSS: −18 vs. −12 points), reinforcing the hypothesis that normalizing substrate availability is a key driver of symptom relief (Figure 9).

## 4. Discussion

Current therapeutic strategies for CFS remain largely symptomatic. This reflects our incomplete understanding of the disease etiology [31,32]. Crucially, in the specific subgroup of patients with co-occurring IBS, gut microbiome dysbiosis acts as a driver of low-grade inflammation [6,7], and the precise causal mechanisms are still debated [8,9]. In this study, we demonstrated that a 12-week intervention with a multi-strain probiotic (De Simone Formulation) was associated with the correction of intestinal dysbiosis. More importantly, this microbiological improvement coincided with a functional shift in tryptophan metabolism that correlated with clinical symptom mitigation.

Baseline analysis confirmed that our patients—specifically recruited with a dysbiotic/IBS phenotype—exhibited reduced alpha diversity and an increase in pro-inflammatory taxa. This was metabolically mirrored by elevated urinary 3-IS. This excessive 3-IS production is a direct marker of bacterial tryptophan catabolism and implies increased intestinal permeability (“leaky gut”) [13,14,15]. Probiotic treatment effectively reduced 3-IS levels to the reference range observed in healthy controls. This suggests that the epithelial barrier was restored and proteolytic bacterial species were suppressed.

**Figure 10:**
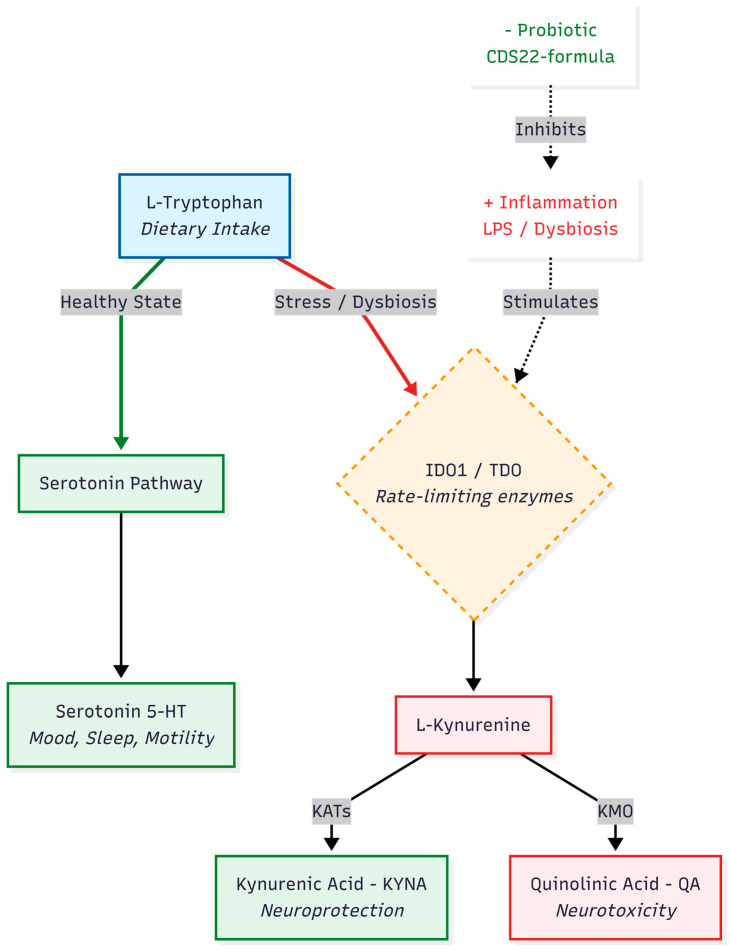
Schematic representation of tryptophan metabolism bifurcation. Under inflammatory conditions driven by gut dysbiosis, tryptophan is preferentially shunted away from the serotonin pathway toward the kynurenine pathway via IDO1 upregulation. The probiotic intervention aims to reduce this inflammatory drive, promoting a shift toward neuroprotective kynurenic acid (KYNA).

Our findings regarding the kynurenine pathway stand in contrast to some prevailing theories. Specifically, our patients exhibited elevated KYN/TRP ratios and high levels of downstream metabolites (XA, QA) at baseline. This indicates a robust activity of indoleamine 2,3-dioxygenase (IDO). Such data contradict the “Metabolic Trap” hypothesis, which assumes a functional block in IDO activity [33]. Instead, our results support a model of chronic, immune-mediated IDO activation, likely driven by persistent dysbiosis [34]. Tryptophan is essentially “stolen” from the serotonin pathway and shunted toward the kynurenine axis, leading to the observed substrate depletion, as illustrated in Figure 10.

The treatment effects were heterogeneous. We saw a robust reduction in xanthurenic acid (XA) and a marked increase in neuroprotective kynurenic acid (KYNA). However, quinolinic acid (QA) levels remained significantly elevated compared to controls. This persistence of QA, despite clear clinical improvement, is puzzling but interpretable. We argue that the absolute concentration of QA is less clinically relevant than its ratio to KYNA [35,36]. Since the intervention increased KYNA by 35%, the KYNA/QA ratio improved by nearly 45%. This suggests a functional neuroprotective blockade: the “antidote” (KYNA) increased enough to neutralize the neurotoxin (QA).

It is also possible that QA clearance is simply slower. QA accumulates in tissues [34]. While gut markers (3-IS) responded rapidly, the persistent urinary QA might reflect a gradual washout of tissue stores or saturation of the QPRT enzyme [37,38].

These metabolic shifts translated into tangible clinical benefits: 87.5% of patients achieved the Minimally Clinically Important Difference in fatigue. However, symptom resolution was not uniform. While fatigue and muscle pain improved, sleep disturbances persisted in the majority of the cohort. This suggests that peripheral metabolic realignment is sufficient to relieve physical fatigue, but central sleep regulation may require more targeted support. We also noted that 15% of patients developed constipation. While Bifidobacteria usually improve transit, alterations in serotonin availability via TRP metabolism changes can evidently affect motility in some individuals.

A critical consideration in interpreting these findings is the distinction between subjective symptom relief and objective metabolic modulation. While the reduction in FSS observed in this open-label trial could theoretically be influenced by the placebo effect or the natural fluctuation of chronic illness, the biochemical data suggest a specific biological mechanism of action. The placebo response typically manifests as a generalized stress reduction, potentially lowering cortisol-mediated TDO activation, but it is unlikely to induce the highly specific pattern of enzymatic shifts observed here—namely, the simultaneous upregulation of kynurenic acid and downregulation of xanthurenic acid. Furthermore, the significant reduction in urinary 3-indoxyl sulfate constitutes a microbiological marker of gut barrier integrity that is independent of host psychology. Thus, while the magnitude of clinical benefit should be viewed with caution given the study design, the consistency of the tryptophan pathway modulation provides strong evidence for a physiological, rather than purely psychogenic, effect of the intervention.

This study has limitations. First, while the sample size (*n* = 40) is relatively modest compared to large-scale population studies, the large effect sizes observed (Cliff’s δ > 0.47; rrb > 0.50) confirm that the study possessed sufficient statistical power to validate the primary metabolic endpoints. Secondly, it was an open-label trial, introducing potential placebo bias. Nevertheless, the rigorous quantification of objective biochemical markers provides a solid physiological basis for the observed changes. Also, measurements were restricted to urine; cerebrospinal fluid analysis would be needed to confirm central neurochemistry changes. Moreover, we strictly excluded confounding factors like high-protein diet [39] or hormonal contraceptives [40], which strengthens the validity of our metabolic findings. Consistent with this rigorous control, the study cohort was restricted exclusively to female participants. While this decision increased internal validity by minimizing the confounding influence of sex hormones on tryptophan metabolism, it limits the generalizability of our findings to the male CFS population. Thirdly, although macronutrient and tryptophan intake were controlled, the study did not strictly monitor dietary FODMAP content. As fermentable carbohydrates can influence gut microbiota activity and IBS symptoms, unmeasured variations in FODMAP intake remain a potential confounding factor. Finally, the durability of the observed metabolic shift after cessation of probiotic supplementation remains unknown. Future longitudinal studies are required to determine whether continuous supplementation is necessary to maintain the neuroprotective KYNA/QA balance.

## 5. Conclusions

Dysbiotic patients with CFS exhibit a metabolic signature of upregulated tryptophan catabolism and a shift toward neurotoxicity. Intervention with the CDS22 formula probiotic restores gut eubiosis and reinstates a neuroprotective balance (KYNA/QA), even if absolute quinolinic acid levels remain elevated. These metabolic corrections correlate with clinical remission, validating the gut–kynurenine axis as a therapeutic target.

## Figures and Tables

**Table 1:** Selected laboratory parameters in CFS patients (*n* = 40) before and after 12 weeks of probiotic intervention.

Parameter	Reference Values	Pre-Treatment (Mean ± SD)	Post-Treatment (Mean ± SD)	*p*-Value
Hematology and Iron Metabolism				
Hemoglobin (g/dL)	12.0–16.0	12.8 ± 0.9	13.4 ± 1.1	0.083
Serum Iron (µg/dL)	60.0–170	93.0 ± 11.1	112.6 ± 12.8	0.046 *
Vitamin B12 (pg/mL)	200–600	441.0 ± 121.2	469.3 ± 103.6	0.654
Metabolic and Renal				
Creatinine (mg/dL)	0.4–1.1	0.8 ± 0.1	0.8 ± 0.2	0.913
eGFR (mL/min)	86–130	88.6 ± 10.5	91.6 ± 6.4	0.756
Vitamin D3 (25-OH-D) (ng/mL)	30.0–50.0	38.8 ± 4.7	42.5 ± 6.4	0.093
Liver and Inflammation				
ALT (U/L)	7.0–30.0	14.9 ± 3.8	15.3 ± 4.5	0.818
AST (U/L)	5.0–35.0	14.3 ± 1.8	13.8 ± 2.1	0.776
C-reactive protein (mg/L)	0.2–5.0	3.1 ± 2.3	3.0 ± 1.4	0.923
Fecal Calprotectin (µg/g)	<50.0	30.9 ± 12.8	23.6 ± 13.1	0.074
Endocrine Profile				
TSH (µIU/mL)	0.4–4.0	3.2 ± 0.9	3.2 ± 0.8	0.932
17-β-Estradiol (pg/mL)	12–300	140.2 ± 39.9	152.7 ± 40.8	0.068
FSH (IU/L)	3.0–12.0	7.2 ± 3.3	7.4 ± 2.2	0.196

Data are presented as mean ± standard deviation. Differences assessed by paired Student’s *t*-test. * *p* < 0.05. Abbreviations: ALT, alanine aminotransferase; AST, aspartate aminotransferase; eGFR, estimated glomerular filtration rate; FSH, follicle-stimulating hormone; TSH, thyroid-stimulating hormone. *p*-values indicate the probability of observing the reported difference under the null hypothesis and were calculated using paired Student’s *t*-test, as specified in the Statistical Analysis section.

**Table 2:** Baseline urinary tryptophan metabolite profile: CFS patients (*n* = 40) vs. healthy controls (*n* = 40).

Variable	Controls Median [IQR]	CFS Median [IQR]	Median Diff. (95% CI)	Effect Size (δ) [95% CI]	q-Value
Gut Microbial Metabolites					
3-IS (mg/gCr)	71.25 [11.68]	88.18 [13.79]	16.9 [11.3, 22.1]	−0.87 (large)	<0.001
3-IS/TRP Ratio	5.59 [1.42]	7.41 [1.43]	1.83 [1.54, 2.47]	−0.85 (large)	<0.001
Kynurenine Pathway					
QA (mg/gCr)	2.65 [1.13]	4.13 [1.34]	1.48 [0.83, 1.96]	−0.69 (large)	<0.001
XA (mg/gCr)	0.60 [0.44]	0.94 [0.17]	0.34 [0.21, 0.47]	−0.74 (large)	<0.001
KYNA (mg/gCr)	2.74 [0.90]	1.93 [0.77]	−0.81 [−1.08, −0.38]	+0.60 (large)	<0.001
KYN (mg/gCr)	0.45 [0.10]	0.49 [0.13]	0.04 [−0.02, 0.13]	−0.22 (small)	0.098
Metabolic Ratios					
KYNA/QA	1.19 [0.90]	0.47 [0.31]	−0.72 [−0.88, −0.40]	+0.71 (large)	<0.001
KYNA/KYN	5.89 [2.70]	3.92 [2.15]	−1.97 [−2.83, −0.85]	+0.42 (medium)	0.002
KYNA/XA	1.43 [2.28]	2.33 [1.03]	0.90 [0.30, 1.32]	−0.34 (medium)	0.012
KYN/TRP	0.03 [0.01]	0.04 [0.02]	0.01 [0.00, 0.02]	−0.34 (medium)	0.011
Substrate and Serotonin					
TRP (mg/gCr)	14.20 [2.58]	12.20 [1.93]	−2.00 [−2.80, −0.65]	+0.55 (large)	<0.001
5-HIAA (mg/gCr)	3.10 [1.30]	3.28 [1.24]	0.18 [−0.30, 0.85]	−0.15 (small)	0.240
5-HIAA/TRP	0.22 [0.10]	0.26 [0.08]	0.04 [0.00, 0.09]	−0.26 (small)	0.052

Data are medians [IQR]. Effect size is Cliff’s delta (δ). Differences assessed by Wilcoxon rank-sum test with Benjamini–Hochberg FDR correction. Abbreviations: 3-IS, 3-indoxyl sulfate; QA, quinolinic acid; XA, xanthurenic acid; KYNA, kynurenic acid; KYN, kynurenine; TRP, tryptophan; 5-HIAA, 5-hydroxyindoleacetic acid. q-values denote false discovery rate–adjusted *p*-values calculated using the Benjamini–Hochberg procedure to account for multiple testing.

**Table 3:** Treatment-induced changes in urinary tryptophan metabolites in CFS patients (*n* = 40 pairs).

Variable	Pre-Treatment (mg/gCr)	Post-Treatment (mg/gCr)	Median Diff. [95% CI]	Effect Size (rrb) [95% CI]	Response (↓/↑/=) *	q-Value
Significant Changes						
XA	0.94 [0.17]	0.70 [0.20]	−0.22 [−0.29, −0.14]	−0.90 (large)	37/2/1	<0.001
3-IS	88.18 [13.79]	76.25 [13.33]	−18.0 [−20.6, −1.6]	−0.80 (large)	27/3/10	<0.001
3-IS/TRP	7.41 [1.43]	6.09 [1.37]	−1.39 [−2.19, −1.01]	−0.75 (large)	35/5/0	<0.001
KYNA	1.93 [0.77]	2.61 [0.49]	+0.60 [0.37, 0.84]	+0.65 (large)	7/33/0	<0.001
KYNA/QA	0.47 [0.31]	0.68 [0.26]	+0.18 [0.10, 0.26]	+0.59 (large)	8/31/1	0.006
5-HIAA	3.28 [1.24]	3.40 [1.43]	+0.23 [0.05, 0.40]	+0.44 (medium)	10/26/4	0.006
KYNA/XA	2.33 [1.03]	3.24 [1.73]	+0.99 [0.42, 1.62]	+0.49 (medium)	10/29/1	0.050
Non-Significant Trends						
QA	4.13 [1.34]	3.92 [1.15]	−0.19 [−0.35, −0.09]	−0.35 (medium)	27/13/0	0.062
TRP	12.20 [1.93]	12.30 [1.18]	+0.30 [−0.10, 0.60]	+0.23 (small)	15/24/1	0.105
KYN	0.49 [0.13]	0.52 [0.21]	+0.02 [−0.07, 0.11]	+0.11 (small)	17/21/2	0.850

Data are medians [IQR]. Differences assessed by paired Wilcoxon signed-rank test. *p*-values adjusted using Benjamini–Hochberg FDR (q-value). Effect size is the rank–biserial correlation (r_rb_). * Response columns indicate number of patients with decrease (↓), increase (↑), or no change (=). Abbreviations: 3-IS, 3-indoxyl sulfate; QA, quinolinic acid; XA, xanthurenic acid; KYNA, kynurenic acid; KYN, kynurenine; TRP, tryptophan; 5-HIAA, 5-hydroxyindoleacetic acid.

**Table 4:** Metabolic normalization: comparison of treated CFS patients (post-Tx) vs. healthy controls.

Variable	Controls (Baseline) Median [IQR]	CFS (Post-Tx) Median [IQR]	Effect Size (δ) [95% CI]	q-Value	Status
Normalized (no significant difference)					
KYNA	2.74 [0.90]	2.61 [0.49]	+0.15 [−0.11, 0.40]	0.258	Normalized
XA	0.60 [0.44]	0.70 [0.20]	−0.26 [−0.50, 0.00]	0.062	Normalized
3-IS	71.25 [11.68]	76.25 [13.33]	−0.27 [−0.50, −0.02]	0.060	Normalized
5-HIAA	3.10 [1.30]	3.40 [1.43]	−0.27 [−0.51, −0.01]	0.060	Normalized
KYN	0.45 [0.10]	0.52 [0.21]	−0.23 [−0.48, 0.02]	0.082	Normalized
Persistent Abnormalities (q < 0.05)					
QA	2.65 [1.13]	3.92 [1.15]	−0.67 (large)	<0.001	Elevated
TRP	14.20 [2.58]	12.30 [1.18]	+0.51 (large)	<0.001	Depressed
KYNA/QA	1.19 [0.90]	0.68 [0.26]	+0.56 (large)	<0.001	Depressed
KYNA/XA	1.43 [2.28]	3.24 [1.73]	−0.41 (medium)	0.006	Elevated
3-IS/TRP	5.59 [1.42]	6.09 [1.37]	−0.37 (medium)	0.013	Elevated

Data are medians [IQR] in mg/gCr. Comparisons assessed by Wilcoxon rank-sum test with Benjamini–Hochberg FDR correction (*q*-value). “Normalized” indicates *q* > 0.05. “Abnormalities” indicate *q* < 0.05. Effect size is Cliff’s delta (δ).

**Table 5:** Longitudinal fatigue severity scale (FSS) outcomes during 12-week treatment.

Time Point	FSS Score (Mean ± SD)	Median [IQR]	Remission (FSS < 36) *n* (%)
Baseline	38.0 ± 8.8	37.5 [31.5–46.0]	18 (45.0%)
Week 4	34.3 ± 8.0	33.0 [28.0–39.2]	23 (57.5%)
Week 8	23.9 ± 6.1	25.5 [18.0–29.0]	39 (97.5%) ***
Week 12	22.7 ± 5.3	23.0 [18.8–26.0]	39 (97.5%) ***

Data are *n* = 40. Statistical significance determined by Friedman test (chi2 = 103.41, *p* < 0.001) with Bonferroni-adjusted paired Wilcoxon post hoc contrasts versus baseline. *** *p* < 0.001.

**Table 6:** Adverse events (AEs) reported during the 12-week probiotic intervention (*n* = 40).

Adverse Event	Incidence *n* (%)	Onset (Week)	Severity	Management/Intervention	Outcome
Constipation (worsening)	6 (15.0%)	2	Mild to Moderate	Osmotic laxatives: Lactulose (2 × 15 mL/d) or Macrogols (2 × sachet/d)	Resolved within 3 days; maintenance dose continued
Transient Bloating	3 (7.5%)	1	Mild	None (spontaneous resolution)	Resolved within 3–7 days
Headache	1 (2.5%)	6	Mild	Analgesics (paracetamol 500 mg, ad hoc)	Resolved within 48 h
Study Discontinuation	0 (0.0%)	—	—	—	—

## Data Availability

The data from this study are available to be shared upon reasonable request.

## Abbreviations

The following abbreviations are used in this manuscript:

3-IS	3-Indoxyl Sulfate
5-HIAA	5-Hydroxyindoleacetic Acid
ALT	Alanine Aminotransferase
AST	Aspartate Aminotransferase
CFQ-11	Chalder Fatigue Scale
CFS	Chronic Fatigue Syndrome
CFU	Colony-Forming Units
CI	Confidence Interval
CRP	C-Reactive Protein
DI	Dysbiosis Index
eGFR	Estimated Glomerular Filtration Rate
FDR	False Discovery Rate
FSH	Follicle-Stimulating Hormone
FSS	Fatigue Severity Scale
GA-map	Genetic Analysis map (Dysbiosis Test)
GC-MS/MS	Gas Chromatography–Tandem Mass Spectrometry
IBS-U	Irritable Bowel Syndrome (Unclassified Subtype)
IDO	Indoleamine 2,3-Dioxygenase
IQR	Interquartile Range
KYN	Kynurenine
KYNA	Kynurenic Acid
LC-MS/MS	Liquid Chromatography–Tandem Mass Spectrometry
MCID	Minimally Clinically Important Difference
NMDA	N-Methyl-D-Aspartate
QA	Quinolinic Acid
SDI	Shannon Diversity Index
TDO	Tryptophan 2,3-Dioxygenase
TRP	Tryptophan
TSH	Thyroid-Stimulating Hormone
XA	Xanthurenic Acid
